# Vector incrimination and transmission of avian malaria at an aquarium in Japan: mismatch in parasite composition between mosquitoes and penguins

**DOI:** 10.1186/s12936-021-03669-3

**Published:** 2021-03-06

**Authors:** Mizue Inumaru, Atsushi Yamada, Misa Shimizu, Ayana Ono, Makiko Horinouchi, Tatsuki Shimamoto, Yoshio Tsuda, Koichi Murata, Yukita Sato

**Affiliations:** 1 Laboratory of Biomedical Science, Department of Veterinary Medicine, College of Bioresource Sciences, Nihon University, 1866 Kameino, Fujisawa, Kanagawa 252-0880 Japan; 2 Niigata City Aquarium Marinepia Nihonkai, 5932-445 Nishifunamicho, Chuo, Niigata 951-8555 Japan; 3 Department of Medical Entomology, National Institute of Infectious Diseases, 1-23-1 Toyama, Shinjuku, Tokyo 162-8640 Japan; 4 Laboratory of Wildlife Science, Department of Animal Resource Sciences, College of Bioresource Sciences, Nihon University, 1866 Kameino, Fujisawa, Kanagawa 252-0880 Japan

**Keywords:** Avian malaria, Blood meal, Japan, Mosquito, Penguin, *Plasmodium*, Transmission cycle

## Abstract

**Background:**

Captive populations of penguins outside of their natural distributions are often maintained in outdoor facilities, such as zoos and aquariums. Consequently, such penguins in captivity are constantly exposed to mosquito vectors and risk of avian malarial infection during their active period from spring to autumn, which can be lethal to these naïve birds. Previous studies have investigated parasite prevalence in mosquitoes or penguins, but simultaneous investigations, which would be crucial to monitor the transmission dynamics and cycle within a facility, have not been done. To identify dominant lineages and trends, multiple-year surveys are recommended.

**Methods:**

Avian malaria parasites (*Plasmodium* spp.) and related haemosporidia were tested in penguins and mosquitoes at an aquarium in Japan through multiple years from 2011 to 2018. Prevalence and dynamics were confirmed, and molecular analyses targeting the protozoal cyt*b* gene were used to reveal the transmission cycle. Blood meals of mosquitoes were also identified using molecular methods.

**Results:**

Parasite detection in penguins tended to fluctuate within an individual. Two *Plasmodium* lineages were consistently detected in mosquitoes that had fed on penguins and wild birds observed around the aquarium. *Plasmodium* lineage CXPIP09 was detected from both mosquitoes and penguins, suggesting active transmission at this facility. However, *Plasmodium cathemerium* PADOM02 was only detected in mosquitoes, which may be due to host, vector or parasite-related factors, or detection methods and their limits. Additionally, *Haemoproteus larae* SPMAG12 was detected from penguins, suggesting active transmission via biting midges.

**Conclusions:**

The mismatch in parasite composition between penguins and mosquitoes shows that multiple aspects such as captive birds, wild birds and vector insects should be monitored in order to better understand and control avian malarial infection within *ex-situ* conservation facilities. Furthermore, morphological analyses would be needed to confirm competency and infection dynamics of avian malaria parasites.

**Supplementary Information:**

The online version contains supplementary material available at 10.1186/s12936-021-03669-3.

## Background

Many wild populations of penguins are at risk of extinction as a result of multiple threats, including pollution, habitat loss, climate change, and infectious diseases. Many species have been designated as endangered species [1]. As part of conservation efforts, ex-situ populations have been established in zoos, aquariums and other breeding facilities throughout the world. Avian malaria, caused by *Plasmodium* parasites, is mainly transmitted by *Culex* mosquitoes [2–5]. Due to their evolutionary background in environments of little contact with vector species, penguins are mostly naïve to avian malaria and many lethal cases of avian malarial infections have been reported in captive penguins [6, 7]. While indoor enclosures can substantially decrease risk of infection by lowering contact with mosquito vectors [6], penguins at most facilities are kept in open-air enclosures throughout the year where mosquito vectors can freely access these captive birds. Mosquitoes can therefore transmit avian malaria parasites among and between captive penguins and wild birds that inhabit surrounding areas [2, 8, 9]. Local mosquitoes and wild birds maintain a constant transmission cycle of avian malaria, which can consequently be transmitted to captive individuals via vectors. Previous studies have indicated that *Culex* mosquitoes are the primary vectors transmitting *Plasmodium* to captive penguins [7–9]. However, most studies have investigated the prevalence in mosquitoes following mortality in penguins, and simultaneous investigations of mosquitoes and penguins have not been done. The role of captive penguins in avian malarial parasite transmission in zoos and aquariums has been poorly investigated. In order to better understand the infection dynamics and transmission within zoo and aquarium facilities, simultaneous investigations of haemosporidia in both penguins and mosquitoes would be needed. Blood-feeding patterns of mosquitoes are an important factor in explaining transmission dynamics of avian malaria parasites and other vector-borne pathogens [10–13]. Blood meal analysis can help to indirectly reveal the transmission cycle within the survey area. Whether blood meal analysis can accurately estimate parasite prevalence in avian hosts has not been assessed.

In Japan, over 3000 penguins are kept at zoos and aquariums, making one of the largest ex-situ populations in the world [14, 15]. Sporadic detections of avian malaria parasites have been reported within the country [16]. However, infection status had not been monitored over multiple years and infection dynamics are unknown. Likewise, parasite prevalence in mosquitoes caught at zoological gardens have been investigated in only a single year [8, 12]. Parasite intensity in host birds is known to fluctuate in relation to circannual changes in both the host and parasite physiology [2, 17–19]. A previous study demonstrated changes in *Plasmodium* lineage composition in *Culex* mosquitoes between years, and also noted that dominant lineages were consistently found in all years [20]. Hence, multiple-year studies are recommended to determine transmission within a study area.

In this study, the prevalence and infection dynamics of avian malaria and related haemosporidian parasites were investigated in both penguins and mosquitoes at an aquarium in northern Japan. Parasite lineages from penguins and mosquitoes were compared across multiple years to determine the transmission dynamics, identify vector species (vector incrimination), and reveal the role of captive penguins within the transmission cycle. Blood meal analysis in mosquitoes was also used to compare results between penguins and mosquitoes in order to evaluate how well such indirect evaluations using mosquitoes reflect the actual host parasite prevalence.

## Methods

### Study site

The study took place at Niigata City Aquarium Marinepia Nihonkai (37°55′ N 139°01′ E), located on the coast of the Sea of Japan. Penguins are the only avian species kept at the facility and are kept in an outdoor enclosure during the whole year, where free access of mosquitoes is possible. The enclosure is connected to a shack, where penguins are able to go in and out freely. Two species, Humboldt penguins (*Spheniscus humboldti*) and southern rockhopper penguins (*Eudyptes chrysocome*), are kept at the aquarium. The facility is surrounded by forested areas. Roughly 20 km southwest of the aquarium is the Sakata wetland, where avian malaria parasite DNA was previously detected from mosquitoes [20]. A freshwater biotope area was created within the facility in 2013 for recreational and educational purposes.

### Penguin samples

Penguins were sampled from 2012 to 2018, mainly during summer and spring, for their routine medical check-ups. No anti-malarial medications were used during the study, and the penguins were in good health. All penguins were born within Japan. In total, 104 Humboldt penguins and two southern rockhopper penguins were sampled. Some individuals were sampled multiple times during the course of the study, although sampling frequency and interval varied between individuals. Blood samples were taken from the metatarsal vein and placed in microtubes containing 70% ethanol. The samples were sent to Nihon University College of Bioresource Sciences Laboratory of Biomedical Science, Fujisawa, Japan and kept at − 20 °C until further processes. Blood smears were not prepared in this study and microscopic observation of parasites was not possible.

Deoxyribonucleic acid (DNA) extraction was done by standard phenol–chloroform method, using Tris–EDTA (ethylene-diamine-tetra-acetic acid) for the final buffer. DNA concentration and quality were confirmed with a spectrophotometer (Nanodrop 1000 or Nanodrop One Microvolume UV–Vis Spectrophotometer; Thermo Fisher Scientific, MA, USA), and the concentration was adjusted to 50 ng/µl. Haemosporidian parasite DNA was detected by a nested polymerase chain reaction (PCR) targeting the partial mitochondrial cytochrome *b* (cyt*b*) gene of the parasite [21]. In brief, the first PCR was carried out with the HaemNFI/HaemNR3 primer set, followed by a second PCR using HaemF/HaemR2 to detect *Plasmodium*/*Haemoproteus* and HaemFL/HaemR2L to detect *Leucocytozoon* parasites. The reaction mixture contained 2 mM MgCL_2_, 0.2 mM deoxynucleotide triphosphate, 10 × ExTaq Mg^2+^ free buffer (TaKaRa, Ohtsu, Japan), 0.625 U Ex-Taq (TaKaRa), 10 µM of each primer, and 50 ng DNA template, making a final volume of 25 µl each. PCR conditions were followed according to protocol [21]. Negative controls containing distilled water instead of DNA was included in every reaction. Positive controls were also included, using 50 ng DNA templates of *Plasmodium gallinaceum* GALLUS01 from an experimentally infected chicken (*Gallus gallus*) and *Leucocytozoon* sp. OTULEM04 from a Sunda scops-owl (*Otus lempiji*) rescued in central Japan. Amplifications were confirmed and positive samples were sequenced following a previously described protocol [22]. The obtained sequences were assembled and compared with sequences in the GenBank database using the Basic Local Alignment Search Tool (BLAST) [23] and the MalAvi database [24]. In the case of any suspicious findings, the sample was re-tested using newly extracted DNA.

### Mosquito samples

Mosquitoes were sampled during the summers (June to October) of 2011 to 2014 and 2018. Females resting within the penguin shack were directly captured in the morning or evening, using film canisters (height 5 cm × diameter 3 cm) or hand-held aspirators. All mosquitoes were morphologically identified to a species [25, 26]. Mosquitoes were placed in − 20 °C until further processes.

For the mosquitoes captured from 2011 to 2014, individuals were randomly selected. Among selected mosquitoes, unfed individuals were removed and only blood-fed (including gravid) individuals were used for analysis. Each individual was separated into head-thorax and abdomen under an Olympus SZ61TR stereo microscope (Olympus, Tokyo, Japan) in order to differentiate the biological status of the parasite (i.e., sporozoites in the salivary gland or gametocytes in the blood meal). Up to five head-thoraxes of the same species were pooled for mosquitoes caught in 2012 to 2014. All abdomen samples from 2012 to 2014 and all samples from 2011 were processed individually. Mosquitoes caught in 2018 were classified into blood-fed or unfed, and all blood-fed individuals were processed individually without dissection.

DNA was extracted from each sample with a REDExtract-N-Amp Tissue PCR kit (SIGMA, St Louis, MO, USA). A nested-PCR targeting the partial cyt*b* gene of *Plasmodium*/*Haemoproteus* parasites was performed, using the DW2/DW4 primer set for first PCR [27] and HAEMNF/HAEMNR for second PCR [28]. The reaction mixture contained 4 mM MgCl_2_, 0.4 mM deoxynucleotide triphosphate, 10 × ExTaq buffer (Mg^2+^ free; Takara, Ohtsu, Japan), 1 U Ex-Taq (Takara), 0.4 µM each primer and 1 µl of template DNA, making the final volume 25 µl each. Positive and negative controls were prepared the same as for bird samples. PCR conditions were followed according to protocol [27, 28]. The remaining processes (i.e., amplification to sequence comparisons) were done following the same methods as for the bird samples.

The abdomens of blood-fed mosquitoes were used to identify the blood-source species. Semi-nested PCRs were performed using the primers Avian-3, Avian-4 and Avian-8 targeting the cyt*b* gene of birds; and Mammalian-1, Mammalian-2 and Mammalian-7 targeting the cyt*b* gene of mammals [29]. The reaction mixture was prepared with the same composition as for parasite detection from mosquitoes, and PCR conditions were followed according to protocol [29]. A negative control containing distilled water instead of DNA was included. All further processes used the same method as above, and the blood-source was identified using the GenBank database [23].

### Statistical analyses

The annual parasite prevalence in penguins and mosquitoes was compared using Fisher’s exact test with the software R version 3.6.3 [30]. For mosquitoes, prevalence (or pooled prevalence) was tested separately for the head-thorax and abdomen. Multiple comparisons with the Bonferroni correction were carried out as a post-hoc test using the package ‘fmsb’ [31]. *Plasmodium* spp. and *Haemoproteus* spp. were tested separately for penguins. If multiple samples were obtained from the same individual in the same year, only one sample was counted. If results differed between the samples (e.g., one positive and one negative), the positive sample was counted. Statistical significance was determined using the 5% significance level. The maximum likelihood estimation of minimum infection rates (MIRs) were calculated using PooledInfRate to estimate the number of infected mosquitoes per 1,000 individuals for pooled samples of varying sizes [32]. For mosquitoes caught in 2011 to 2014, the MIR was calculated separately for head-thorax and abdomen. For mosquitoes caught in 2011, an individual was considered positive if either the head-thorax or abdomen was positive, and the total MIR was calculated.

## Results

### Prevalence and genetic diversity of haemosporidian lineages in penguins

**Fig. 1:**
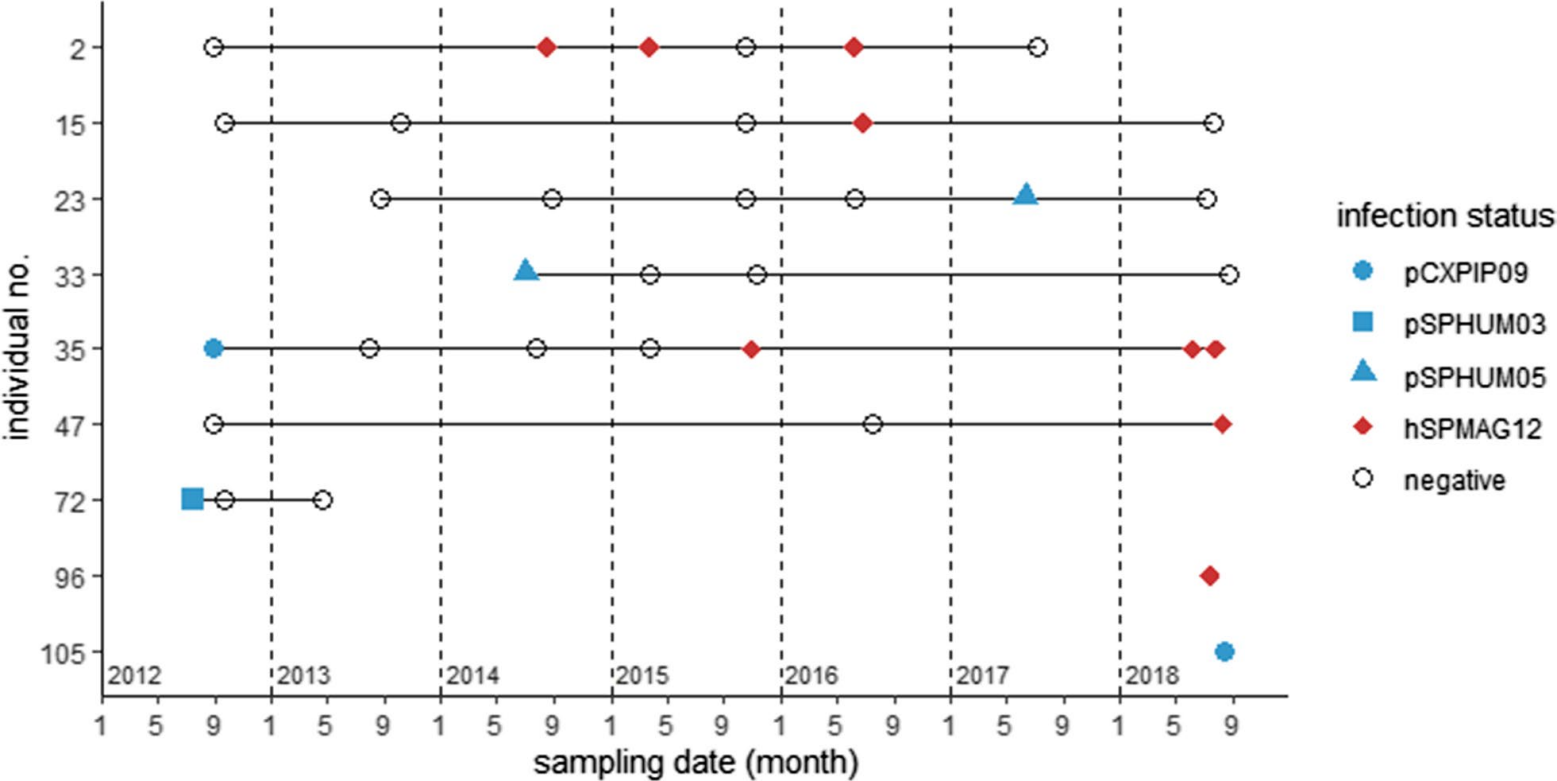
Parasite detection dynamics in PCR-positive penguins. Blue marks: *Plasmodium* spp. detection; red marks: *Haemoproteus* spp. detections; open circles: negative

Over the course of 7 years, a total of 268 blood samples were obtained from 106 individuals. Nine of the tested penguins (8.49%) were positive for *Plasmodium* or *Haemoproteus* in at least one sampling time point (Fig. 1). Annual prevalence ranged from 0 to 4.26% for *Plasmodium* and 0 to 6.52% for *Haemoproteus* (Table 1). No significant difference was detected among years for both genera (Fisher’s exact test: *Plasmodium* spp. *p* = 0.65; *Haemoproteus* spp. *p* = 0.35). Infection status tended to fluctuate in the positive individuals among years, going from positive to negative and vice versa. Of the positive individuals, *Plasmodium* spp. and *Haemoproteus* spp. were detected from five individuals each. In one individual (no. 35), *Plasmodium* sp. CXPIP09 was initially detected, but *Haemoproteus larae* SPMAG12 was detected in later samples. Three *Plasmodium* lineages were detected, including one newly detected lineage. The new lineage, SPHUM05 was deposited to GenBank and MalAvi (see Additional file 1: Table S1 for information on the detected lineages). One lineage was identical to Caleu1, previously detected from a streaked shearwater (*Calonectris leucomelas*) of Japan (accession no. AB601429). This lineage was newly named SPHUM03 according to MalAvi nomenclature and was deposited to GenBank and MalAvi. *Haemoproteus larae* SPMAG12 was the only detected *Haemoproteus* lineage. *Leucocytozoon* was not detected in this study.

**Table 1:** Annual prevalence of *Plasmodium* and *Haemoproteus* by PCR in penguins

Year	Number of samples	*Plasmodium*		*Haemoproteus*	
		Number positive	Prevalence (%)	Number positive	Prevalence (%)
2012	47	2	4.26	0	0
2013	33	0	0	0	0
2014	33	1	3.03	1	3.03
2015	35	0	0	2	5.71
2016	45	0	0	2	4.44
2017	29	1	3.45	0	0
2018	46	1	2.17	3	6.52

Duplicate samples from the same individual in the same year have been removed

### Prevalence and genetic diversity of haemosporidian lineages in vectors

**Fig. 2:**
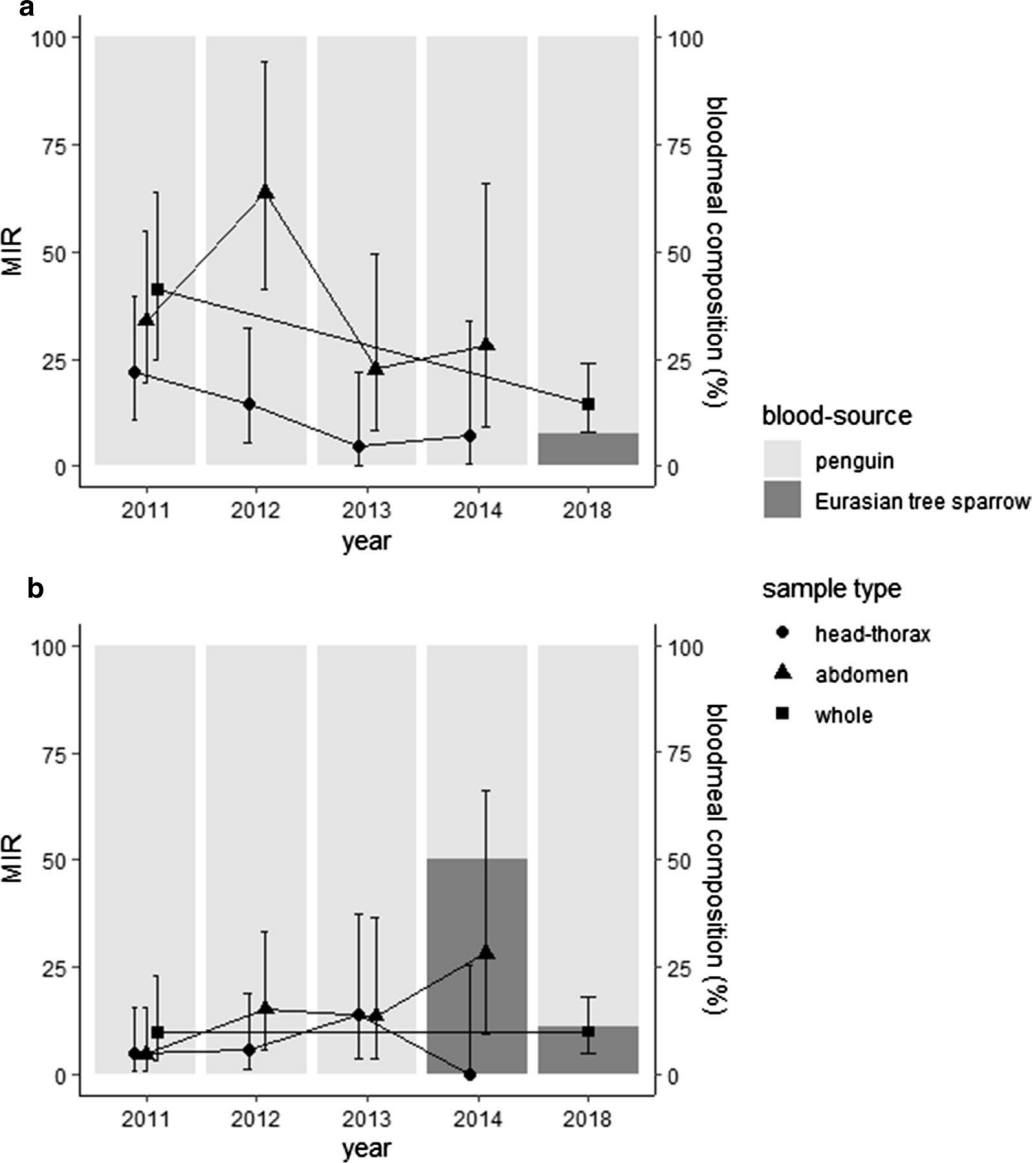
Minimum infection rate (MIR) of *Plasmodium* in blood-fed *Culex pipiens* group and blood meal composition of *Plasmodium*-positive individuals. *Plasmodium* sp. CXPIP09 (**a**) and *Plasmodium cathemerium* PADOM02 (**b**). MIR is shown by each sample type, and error bars represent ± 95% of confidence interval

*Culex pipiens* group and *Culex tritaeniorhynchus* were caught in all sampling years (Table 2). Some years, *Aedes albopictus* was caught in small numbers, and one *Culex bitaeniorhynchus* was caught in 2018. *Plasmodium* were detected from *Cx. pipiens* group every sampling year. *Plasmodium* spp. CXPIP09 and PADOM02 were detected from *Cx. pipiens* group in all years (Table 3, Fig. 2). The prevalence of these lineages did not significantly differ among years (Fisher’s exact test: CXPIP09 pooled prevalence in head-thorax *p* = 0.09; CXPIP09 prevalence in abdomen *p* = 0.08; PADOM02 prevalence in abdomen *p* = 0.13). For the pooled PADOM02 prevalence in head-thorax, Fisher’s exact test initially resulted in a significant *p*-value (*p* = 0.01). However, the Bonferroni-corrected pairwise Fisher’s test revealed no significant results (multiple comparison Fisher’s test with Bonferroni correction: 2011 *vs* 2013 *p* = 0.52; 2011 *vs* 2013 *p* = 0.06; all others *p* = 1.00). The MIR of these two lineages, as well as the overall MIR, tended to be higher in abdomen samples compared to head-thorax samples. Analysis of mosquitoes caught in 2011 reveal that many individuals were positive for only head-thorax or abdomen (Table 3). Furthermore, two individuals were positive for different lineages in the head-thorax and abdomen. *Plasmodium* sp. SPHUM05 was also detected from head-thorax of three *Cx. pipiens* group (Table 3). *Plasmodium*-positive *Cx. tritaeniorhynchus* were caught only in 2018, and the lineages CXPIP09 and GALLUS02 were detected.

**Table 2:** Number of female mosquitoes collected and tested, including blood meal analysis results

Year	Species	Caught	Tested	Blood meal		
				Penguin	Wild bird	Unknown
2011	*Cx. pipiens* group	1013	413^a^	298	1	114
	*Cx. tritaeniorhynchus*	19	17	8	0	9
	*Ae. albopictus*	9	1	–	–	–
	sub-total	1041	431	306	1	123
2012	*Cx. pipiens* group	3652	329^a^	239	2	88
	*Cx. tritaeniorhynchus*	2	2	2	0	0
	*Ae. albopictus*	4	1	–	–	–
	sub-total	3658	332	241	2	88
2013	*Cx. pipiens* group	783	220^a^	159	2	59
	*Cx. tritaeniorhynchus*	26	26	24	0	2
	sub-total	809	246	183	2	61
2014	*Cx. pipiens* group	536	142^a^	109	2	31
	*Cx. tritaeniorhynchus*	1	1	0	0	1
	sub-total	537	143	109	2	32
2018	*Cx. pipiens* group	932	901^ab^	20	2	0
	*Cx. tritaeniorhynchus*	30	30^ab^	2	0	0
	*Cx. bitaeniorhynchus*	1	1	–	–	–
	*Ae. albopictus*	2	0	–	–	–
	sub-total	965	932	22	2	0
	total	7010	2084	867	9	304

^a^Includes *Plasmodium* positive individuals and/or pools

^b^Bloodmeal investigated for only *Plasmodium*-positive mosquitoes

**Table 3:** PCR results of *Plasmodium* spp. detection and Minimum Infection Rate (MIR) in Mosquitoes

Year	Species	Total specimens	Head-thorax			Abdomen		Whole body		Lineages^d^		
			No. pools	No. positive	MIR (± 95% CI)	No. positive	MIR (± 95% CI)	No. positive	MIR (± 95% CI)	CXPIP09	PADOM02	Others
2011^a^	*Cx. pipiens* group	413	413	13	31.48 (17.67–51.83)	16	38.74 (23.15–60.78)	22	53.27 (34.58–78.19)	ht(2), ab(7), ht-ab(6)	ht(1), ab(1)	htCXPIP09-abPADOM02(1) htPADOM02-abCXPIP09(1) htSPHUM05(2)
2012^b^	*Cx. pipiens* group	329	63	7	20.79 (9.27–40.63)	26	79.03 (53.44–111.99)	–	–	ht(5), ab(21)	ht(2), ab(5)	
2013^b^	*Cx. pipiens* group	220	50	5	23.56 (8.84–51.47)	8	36.36 (17.14–67.7)	–	–	ht(1), ab(5)	ht(3), ab(3)	htSPHUM05(1)
2014^b^	*Cx. pipiens* group	142	43	1	7.05 (0.41–33.79)	8	56.34 (26.68–103.88)	–	–	ht(1), ab(4)	ab(4)	
2018^c^	*Cx. pipiens* group	901	–	–	–	–	–	22	24.42 (15.77–36.11)	whole(13)	whole(9)	
	*Cx. tritaeniorhynchus*	30	–	–	–	–	–	2	33.33 (1.92–150.09)	whole(1)		GALLUS02(1)

ht: detection from the head-thorax; ab: detection from the abdomen

^a^All mosquitoes were tested individually upon dissection

^b^Head-thorax were pooled and abdomen were individually tested

^c^All mosquitoes were tested individually without dissection

^d^Numbers in parentheses represent numbers of positive samples

### Blood meal analyses

Blood hosts of the captured mosquitoes were mostly penguins, but a few *Cx. pipiens* had fed on wild birds, including the Japanese white-eye (*Zosterops japonicus*), oriental greenfinch (*Chloris sinica*), and Eurasian tree sparrow (*Passer montanus*) (Table 2). Most *Plasmodium*-positive *Cx. pipiens* had fed on penguins. Meanwhile, some individuals positive for the lineages CXPIP09 and PADOM02 had fed on Eurasian tree sparrows (Fig. 2). Both *Plasmodium*-positive *Cx. tritaeniorhynchus* had fed on penguins.

## Discussion

### Infection dynamics in penguins

A total of 9 penguins were positive for avian haemosporidia. All individuals were in good health, exhibiting no observable symptoms. Previous studies have reported lethal cases of avian malaria in captive penguins [6, 7]. Differences in virulence have been suggested between cyt*b* lineages and MSP1 types [16, 33]. Lineages detected in this study may potentially be less virulent compared to other lineages, although further monitoring would be needed to confirm. While no positive individuals were detected in some years, the parasite prevalence of both genera did not significantly differ annually. Therefore, both *Plasmodium* and *Haemoproteus* are thought to be maintained within the penguin population at low levels. For some individuals, samples were initially negative, but later samples were positive. Such cases may possibly reflect newly gained infections. Meanwhile, in many individuals, the infection status went from positive to negative (nos. 33 and 72) or fluctuated (nos. 2, 15, 23, 35). The parasite intensity is generally known to increase in the spring and summer (i.e., spring relapse) and decrease, or even disappear from the bloodstream, in the autumn and winter [2, 17–19]. While PCR is a highly sensitive tool for detection of DNA, extremely low parasitaemia may possibly be below the detection limit. In a recent study, PCRs of *Haemoproteus*-infected penguin DNA were negative using 50 ng DNA template, but positive using 200 ng DNA [22]. All PCR tests in this study were done using 50 ng of DNA template. Circannual changes could have possibly caused a fluctuation in parasitaemia, causing some samples to become negative due to the PCR detection limit. The use of higher DNA concentration may be needed to monitor the infection status of these penguins more precisely. Morphological detections and parasitaemia counts using blood smears would also help to understand causes of these fluctuating results. Note that for one individual (no. 35), the parasite lineage changed from *Plasmodium* sp. CXPIP09 to *Haemoproteus* sp. SPMAG12. This individual may have been newly infected with SPMAG12 during the study. However, co-infections are difficult to detect, especially when co-infected with *Plasmodium* and *Haemoproteus*, and one parasite may be favoured over the other using PCR [34–36]. In relation to the circannual fluctuation of parasitaemia, detectability may also fluctuate between these lineages during co-infection. Therefore, it was not possible to distinguish new detections or fluctuations of parasitaemia in this study.

### Infection dynamics in mosquitoes

*Plasmodium*-positive *Cx. pipiens* group were detected in all years of sampling, and the prevalence of both CXPIP09 and PADOM02 was relatively stable between years. These results suggest that *Cx. pipiens* group is the primary vector of avian malarial parasites at this aquarium and that infection dynamics are relatively stable among years. The MIR tended to be higher in the abdomen compared to the head-thorax, presumably due to the presence of the gametocytes within the blood meal. In general, positive detections of avian malaria from the head-thorax suggest the presence of sporozoites in the salivary gland, which are necessary for the transmission of avian malaria from mosquitoes to host birds. Similarly, positive detections from the abdomen suggest the presence of gametocytes in the blood meal or oocysts in the midgut [2]. Digestion of blood in the abdomen takes approximately 2 to 5 days, although the period differs by species and temperature [37–40]. *Plasmodium* sporozoites reach the salivary gland of the mosquito in 4 to 14 days post-blood meal, also differing by factors including parasite species, intensity and temperature [2, 39, 41]. Considering the duration and timing of blood meal digestion and parasite development, parasite detections from the head-thorax of blood-fed mosquitoes is thought to be due to the presence of sporozoites obtained from previous blood meals. Hence, mosquitoes would have had to take a second blood meal, while detection in the abdomen requires only one blood meal. However, it may be possible that parasite detections in the head-thorax are due to remnant blood meal within the head-thorax [11]. If conditions are favourable, a mosquito can complete digestion, oviposition and take a second blood meal within 5 to 7 days of the first blood meal [38, 41]. Oocysts can persist in the midgut for up to 16 to 24 days post-blood meal [11, 42, 43], which means that detections from blood-fed abdomens could also potentially be due to oocysts from the first blood meal. Meanwhile, in two individuals, different parasite lineages were detected from the head-thorax and abdomen. It is expected that the same lineage would be detected if remnant blood meal was in the head-thorax. Such cases suggest that the lineage in the head-thorax was obtained in the first blood meal, and the lineage in the abdomen was obtained by the second blood meal.

*Plasmodium* parasites were detected from *Cx. tritaeniorhynchus* in only 2018. Far fewer *Cx. tritaeniorhynchus* were caught each year compared to *Cx. pipiens* group, and this may have resulted in the low detection rate. These two species have different preferences in resting locations, as *Cx. tritaeniorhynchus* prefers outdoor environments, while *Cx. pipiens* group prefers indoor environments [39, 44, 45]. All mosquito collection in this study was done inside the penguin shack. Outdoor collections of mosquitoes may result in an increased collection of *Cx. tritaeniorhynchus*, which may offer a more accurate estimation of parasite prevalence in this species.

### Transmission cycle

In total, three and four *Plasmodium* lineages were detected from penguins and mosquitoes, respectively. Of these, CXPIP09 and SPHUM05 were the only shared lineages between penguins and mosquitoes. CXPIP09 is widely distributed in Japan and has thus far been detected only in Japan. It has been previously detected from both mosquitoes and wild birds (see Additional file 1: Table S1 for detailed information on the detected lineages, including previous detections). Both sporozoites and oocysts of CXPIP09 have been confirmed in *Cx. pipiens* group, verifying that this mosquito species is a competent vector [5]. This lineage was detected from mosquitoes in all sampling years, strongly suggesting that this lineage is maintained at the study site. Despite the constant detection and stable MIR among mosquitoes, CXPIP09 was only detected from two penguins throughout the study. This could possibly be due to the limited detectability of sub-microscopic parasitaemia, as discussed above. Even when parasitaemia is sub-microscopic, mosquitoes are capable of developing oocysts [46]. Another possibility could be that infected penguins are more attractive to mosquitoes and therefore get bitten more often. Host birds with chronic infections have been shown to attract significantly more mosquitoes compared to birds with acute infection and uninfected birds [47–50]. Both possibilities are assuming that penguins are competent hosts of CXPIP09, which has not yet been confirmed due to the absence of blood smears. While most of the CXPIP09-infected mosquitoes had fed on penguins, some had fed on wild Eurasian tree sparrows. This suggests that *Cx. pipiens* group can carry CXPIP09 between penguins at the facility and wild birds that inhabit the surrounding areas. These results are in agreement with previous studies that have established *Plasmodium* spp. in captive penguins are acquired from wild birds in the surroundings [7, 9]. Meanwhile, if penguins are competent hosts, they may also act as reservoirs for transmission. To our knowledge, CXPIP09 was detected from *Cx. tritaeniorhynchus* for the first time. However, the mosquito was blood-fed and its vector competency remains unknown.

*Plasmodium* sp. SPHUM05 was detected from the head-thorax of three *Cx. pipiens* group and blood of two penguins. This lineage was detected for the first time and information is limited. However, as all penguins were born within Japan, infections had to have occurred in Japan although the natural host has not yet been identified. Detection from the head-thorax of mosquitoes suggests the possibility that *Cx. pipiens* group may be competent vectors of this lineage, but further investigations are necessary.

The lineage PADOM02 is widespread and has been detected from mainly passeriform birds of Europe, Asia, North America, and North Africa (see Additional file 1: Table S1). It has also been detected from multiple mosquito species of Japan, including *Cx. pipiens* group. A recent study revealed that this lineage belongs to the morphological species *Plasmodium cathemerium* and that *Cx. pipiens* group are competent vectors of this lineage [51]. In this study, this lineage was constantly detected from mosquitoes suggesting that this lineage is also a dominant lineage maintained within the area. Meanwhile, PADOM02 was not detected from penguins. However, blood meal analysis revealed that positive mosquitoes had mostly fed on penguins and some on Eurasian tree sparrows. This mismatch can be explained by three hypotheses. First, the parasitaemia in penguins may be too low for PCR detection, as discussed above. As previous detections of this lineages have been from predominantly passeriform birds, this hypothesis seems rather improbable. Observations of blood smears and PCR using higher DNA concentration would help to further investigate this hypothesis. Second, mosquitoes may have taken their first blood meal from an infected wild bird and second blood meal from an uninfected penguin after oviposition. As mentioned above, in favourable conditions, mosquitoes can take a second blood meal within 7 days of the first blood meal [38, 41], while oocysts and sporozoites can persist for up to 24 days post-blood meal [11, 42, 43]. If the first blood meal was from a PADOM02-infected wild bird and the second from an uninfected penguin, the persisting parasite may have been detected along with blood meal from the second host. Third, mosquitoes may have subsequently taken blood meals from multiple hosts. If sufficient amounts of blood are not obtained in the first blood meal, mosquitoes can take multiple meals from different individuals to achieve sufficient amounts for oviposition [52]. The latter two hypotheses in combination with the lack of detection from penguins suggest that penguins are incompetent hosts of PADOM02 and are refractory. Avian parasites have been detected from vectors that had fed on non-avian hosts [53, 54], suggesting that such mismatches are not necessarily rare. Blood meal analysis is a useful tool especially when investigating vector host range and preferences. However, the blood host should not be directly interpreted as the avian host of the parasite. Meanwhile, detection of Eurasian tree sparrow DNA from PADOM02-infected mosquitoes is not surprising, as Eurasian tree sparrows are likely to be natural hosts of PADOM02, considering previous detections from this species [55].

*Plasmodium juxtanucleare* (GALLUS02) was detected from one *Cx. tritaeniorhynchus* that had fed on penguins. This species has a wide distribution across the Neotropical, Ethiopian and Oriental regions and primary hosts are members of the Phasianidae family, including both wild and captive birds [2, 56–58] (see Additional file 1: Table S1 for information on GALLUS02). *Culex* mosquitoes have been confirmed to be vectors of this species, including *Cx. tritaeniorhynchus* [57]. GALLUS02 was previously detected in one *Cx. pipiens* group caught in Sakata wetland of Niigata [20], and may persist within the area in low levels. While it is unknown whether penguins are competent hosts of GALLUS02, a previous study suggests that *P. juxtanucleare* may be associated to the mortality of black-footed penguins (*Spheniscus demersus*) [59]. Continued monitoring of parasite prevalence and close monitoring of health status for penguins should be carried out.

Two lineages, *Plasmodium* sp. SPHUM03 and *Haemoproteus larae* SPMAG12, were detected solely from penguins. The former has been detected previously from a streaked shearwater of Niigata (accession no. AB601429) and may presumably be transmitted between captive penguins and wild birds via mosquitoes. Vector species of this lineage are still unknown. In this study, numerous *Cx. pipiens* were sampled and investigated for avian haemosporidia. However, mosquito species other than *Cx. pipiens* group were far less investigated due to the limited sample size, and such species may possibly be involved in the transmission of this lineage. The latter was recently identified as *Haemoproteus larae* and is suggested to be transmitted between captive penguins and wild gulls via biting midges [22]. The aquarium in this study is located on the coast where gulls are frequently seen. Biting midges have been seen within the penguin shack (pers. comm.). Like other facilities in Japan, *Haemoproteus larae* (SPMAG12) may be regularly transmitted within and around this facility.

## Conclusions

Avian *Plasmodium* was detected by PCR from both mosquitoes and penguins at an aquarium in Japan. Some lineages suggest active transmission between wild birds and penguins via mosquitoes, supported by blood meal analysis results. Meanwhile, PADOM02 was only detected from mosquitoes, including many that had fed on penguins. Such mismatching results may be due to host, vector or parasite-related factors, or detection methods and their limits. Penguins may possibly be competent for some local lineages, and infected individuals may act as reservoirs to drive the local transmission. Meanwhile, penguins may be refractory to other lineages and transmission of these lineages may be limited to the local avian and vector fauna. Furthermore, analyses on blood meal and parasite prevalence should be carefully assessed, as blood hosts may not always be the parasite host. *Haemoproteus larae* (SPMAG12) was detected from penguins, indicating transmission between penguins and biting midges at this facility. To further understand the infection dynamics and transmission cycle of avian haemosporidia at this facility, the combination of molecular and morphological methods for detection of parasites would be needed. This study emphasizes the need to investigate all aspects such as captive birds, wild birds and vector insects to better understand the transmission cycle within ex-situ conservation facilities.

## Supplementary Information

**Additional file 1: Table S1**. Detailed information on genetic lineages of avian malarial parasites detected in this study, including previous detections.

## Data Availability

All data generated or analysed during the current study are included in this published article.

## Abbreviations

BLAST: Basic Local Alignment Search Tool
DNA: Deoxyribonucleic acid
EDTA: Ethylene-diamine-tetra-acetic acid
MIR: Minimum infection rate
PCR: Polymerase chain reaction
cyt*b*: Cytochrome *b*
