# Supplementary material for: Vector incrimination and transmission of avian malaria at an aquarium in Japan: mismatch in parasite composition between mosquitoes and penguins
Source: Malar J. 2021 Mar 6;20:136. doi: 10.1186/s12936-021-03669-3 (PMC7937252; doi:10.1186/s12936-021-03669-3)
Supplement: Supplementary file 1 — Additional file 1: Table S1. Detailed information on genetic lineages of avian malarial parasites detected in this study, including previous detections. [file 12936_2021_3669_MOESM1_ESM.docx]

**Table S1**. Detailed information on genetic lineages of avian malarial parasites detected in this study, including previous detections.

| genetic  lineage | morpho species | accession no. | previously identified hosts | |  | previously identified vectors | |
| --- | --- | --- | --- | --- | --- | --- | --- |
|  |  |  | species^bc^ | locality^d^ |  | species^b^ | locality^d^ |
| CXPIP09 | *Plasmodium* sp. | AB474376 | *Larus argentatus*(R)  *Spheniscus humboldti*(C)  *Ardea cinerea*(R)  *Corvus corone*(R)  *Corvus macrorhynchos*(R)  *Cyanopica cyanus*(R) | Japan [55,61] |  | *Culex pipiens pallens*  *Culex pipiens* form *molestus*  *Culex pipiens* group  *Culex inatomii*  *Culex sasai*  *Lutzia vorax* | Japan [5,8,11,12,20,60,62,63] |
| GALLUS02 | *Plasmodium juxtanucleare* | AB250415 | *Crossoptilon crossoptilon*(C)  *Eudyptes* *chrysocome*(C)  *Streptopelia* *orientalis*(W) | Japan [56] |  | *Culex pipiens pallens* | Japan [20] |
| PADOM02 | *Plasmodium cathemerium* | DQ058612 | *Phasianus colchicus* (W)  *Calidris canutus* (W)  *Corvus corone* (W)  *Luscinia svecica* (W)  *Passer domesticus* (W)  *Passer montanus* (C,W)  *Motacilla flava* (W)  *Anthus hodgsoni* (W)  *Carpodacus erythrinus* (W)  *Emberiza citrinella* (W)  *Emberiza godlewskii* (W) | South Korea [72]  United States [64]  Japan [72]  Norway [72]  Spain [65,66,68]  France [69,70]  Romania [68]  Turkey [68]  Egypt [51,68]  Japan [55]  Spain [67]  South Korea [72]  Czech Republic [71]  Russia [73]  New Zealand [69]  China [74] |  | *Culex pipiens* group  *Aedes albopictus*  *Culex pipiens pallens*  *Culex pipiens* group  *Culex bitaeniorhynchus*  *Culex inatomii*  *Lutzia vorax* | Switzerland [75,76]  Egypt [51]  Japan [8,12,20,60,61,62] |
| SPHUM03 | *Plasmodium* sp. | LC596948 | *Calonectris leucomelas* (W) | - |  | - | - |
| SPHUM05^a^ | *Plasmodium* sp. | LC596949 | - | - |  | - | - |
| SPMAG12 | *Haemoproteus larae* | AB604310 | *Spheniscus demersus* (C)  *Spheniscus magellanicus* (C) | Japan [22] |  | - | - |

^a^ Newly detected lineage.

^b^ Host and vector species were listed according to the MalAvi database (accessed January 2021).

^c^ The host environments are denoted by the following abbreviations: captive (C), rescued (R), wild (W).

^d^ Only published localities are listed.

60. Shirotani A, Shibata A, Ejiri H, Sato Y, Tsuda Y, Hatakeyama Y, et al. [Detection of avian malaria DNA from mosquitoes in Kanagawa in Japan] (in Japanese). J Jpn Vet Med Assoc. 2009;62:73–9.

61. Tanigawa M, Sato Y, Ejiri H, Imura T, Chiba R, Yamamoto H, et al. Molecular identification of avian haemosporidia in wild birds and mosquitoes on Tsushima Island, Japan. J Vet Med Sci. 2013;75:319–26.

62. Kim KS, Tsuda Y. Seasonal changes in the feeding pattern of *Culex pipiens pallens* govern the transmission dynamics of multiple lineages of avian malaria parasites in Japanese wild bird community. Mol Ecol. 2010;19:5545–54.

63. Kim KS, Tsuda Y, Yamada A. Bloodmeal identification and detection of avian malaria parasite from mosquitoes (Diptera: Culicidae) inhabiting coastal areas of Tokyo Bay, Japan. J Med Entomol. 2009;46:1230-4.

64. D’Amico VL, Baker AJ. A rare case of *Plasmodium* (*Haemamoeba*) *relictum* infection in a free-living Red Knot (*Calidris canutus rufa*, Scolopacidae). J Ornithol. 2010;151:951-4.

65. Ferraguti M, Martínez-de la Puente J, Bensch S, Roiz D, Ruiz S, Viana DS, et al. Ecological determinants of avian malaria infections: an integrative analysis at landscape, mosquito and vertebrate community levels. J Anim Ecol. 2018;87:727-40.

66. Garcia-Longoria L, Marzal A, De Lope F, Garamszegi L. Host-parasite interaction explains variation in the prevalence of avian haemosporidians at the community level. PLoS One. 2019;14:e0205624.

67. Hellgren O, Waldenström J, Perez-Tris J, Szöll E, Si Ö, Hasselquist D, et al. Detecting shifts of transmission areas in avian blood parasites—a phylogenetic approach. Mol Ecol. 2007;16:1281-90.

68. Marzal A, Ricklefs RE, Valkiūnas G, Albayrak T, Arriero E, Bonneaud C, et al. Diversity, loss, and gain of malaria parasites in a globally invasive bird. PLoS One. 2011;6:e21905.

69. Bonneaud C, Pérez-Tris J, Federici P, Chastel O, Sorci G. Major histocompatibility alleles associated with local resistance to malaria in a passerine. Evolution. 2006;60:383-9.

70. Loiseau C, Zoorob R, Robert A, Chastel O, Julliard R, Sorci G. *Plasmodium* *relictum* infection and MHC diversity in the house sparrow (*Passer domesticus*). Proc Biol Sci. 2011;278:1264-72.

71. Synek P, Albrecht T, Vinkler M, Schnitzer J, Votýpka J, Munclinger P. Haemosporidian parasites of a European passerine wintering in South Asia: diversity, mixed infections and effect on host condition. Parasitol Res. 2013;112:1667-77.

72. Beadell JS, Ishtiaq F, Covas R, Melo M, Warren BH, Atkinson, CT, et al. Global phylogeographic limits of Hawaii's avian malaria. Proc Biol Sci. 2006;273:2935-44.

73. Palinauskas V, Iezhova TA, Križanauskienė A, Markovets MY, Bensch S, Valkiūnas G. Molecular characterization and distribution of *Haemoproteus minutus* (Haemosporida, Haemoproteidae): a pathogenic avian parasite. Parasitol Int. 2013;62:358-63.

74. Liu B, Deng Z, Huang W, Dong L, Zhang Y. High prevalence and narrow host range of haemosporidian parasites in Godlewski's bunting (*Emberiza godlewskii*) in northern China. Parasitol Int. 2019;69:121-5.

75. Glaizot O, Fumagalli L, Iritano K, Lalubin F, Van Rooyen J, Christe P. High prevalence and lineage diversity of avian malaria in wild populations of great tits (*Parus major*) and mosquitoes (*Culex pipiens*). PLoS ONE. 2012;7:e34964.

76. Lalubin F, Delédevant A, Glaizot O, Christe P. Temporal changes in mosquito abundance (*Culex pipiens*), avian malaria prevalence and lineage composition. Parasit Vectors. 2013;6:307.
